# Psychometric Properties of the Chinese Version of the Brief Borderline Symptom List in Undergraduate Students and Clinical Patients

**DOI:** 10.3389/fpsyg.2018.00605

**Published:** 2018-04-27

**Authors:** Huihui Yang, Xiaoxia Lei, Mingtian Zhong, Qi Zhou, Yu Ling, Martin Jungkunz, Jinyao Yi

**Affiliations:** ^1^Medical Psychological Center, The Second Xiangya Hospital, Central South University, Changsha, China; ^2^Center for Studies of Psychological Application, School of Psychology, South China Normal University, Guangzhou, China; ^3^Department of Business Administration, School of Business, Sun Yat-sen University, Guangzhou, China; ^4^Education Institute, Hunan Agricultural University, Changsha, China; ^5^Central Institute of Mental Health, Clinic of Psychosomatic and Psychotherapeutic Medicine, Medical Faculty Mannheim, Heidelberg University, Mannheim, Germany; ^6^Central Institute of Mental Health, Institute for Psychiatric and Psychosomatic Psychotherapy/Psychosomatic Medicine and Psychotherapy, Medical Faculty Mannheim, Heidelberg University, Mannheim, Germany; ^7^Medical Psychological Institute, Central South University, Changsha, China

**Keywords:** borderline personality disorder, borderline symptom list, factor structure, reliability, validity

## Abstract

The brief version of the Borderline Symptom List (BSL-23) is a self-rated scale developed from the initial 95-item version of Borderline Symptom List (BSL-95). The current study aimed to evaluate the psychometric properties of the Chinese version of the BSL-23. A total of 570 undergraduate students and 323 clinical patients completed the BSL-23, the borderline subscale of the Personality Diagnostic Questionnaire (PDQ-4+), the Center for Epidemiologic Studies Depression Scale (CES-D), the Barratt Impulsiveness Scale, 11th version (BIS-11), the Childhood Trauma Questionnaire (CTQ) and the Attachment Style Questionnaire (ASQ). A Confirmatory Factor Analysis (CFA) was conducted to test the one-factor structure of the BSL-23. Cronbach’s alpha, Omega coefficient, Split-Half coefficient, Mean Inter-Item Correlation (M_IC_) and test-retest reliability were also measured. The correlations between the BSL-23 and other psychological variables were used to assess criterion-related validity and convergent validity. Participants who scored ≥ 5 on the borderline subscale of the PDQ-4+ were placed into the borderline personality disorder (BPD) screening-positive group, while the others were placed into the screening-negative group. Independent sample *t*-tests were performed to examine the differences in BSL-23 scores between the BPD screening-positive group and the BPD screening-negative group. The CFA results supported the one-factor structure of the BSL-23 in both samples. The internal consistency was high both in the undergraduate sample (Cronbach’s α = 0.93, Omega = 0.95, Split-Half coefficient = 0.89, M_IC_ = 0.38) and the clinical sample (Cronbach’s α = 0.97, Omega = 0.97, Split-Half coefficient = 0.96, M_IC_ = 0.56). The test-retest reliability within 2 weeks was 0.62. The BSL-23 displayed moderate to high correlations with the PDQ-4+-Borderline subscale, the CES-D, the BIS-11, the CTQ and the ASQ (*r* = 0.35 – 0.70). In addition, the BSL-23 discriminated between the BPD screening-positive and the BPD screening-negative participants, and also between the patient sample and undergraduate sample. In conclusion, the Chinese version of the BSL-23 has satisfactory psychometric properties to assess BPD symptoms.

## Introduction

Borderline personality disorder (BPD) is a severe mental disorder characterized by deficits in emotion regulation, impulse control, interpersonal relationships and self-perception (APA, 2013). Emotional dysregulation is a typical symptom of BPD. Patients are more sensitive to emotional stimuli (Winter, 2016) and frequently use maladaptive emotion regulation strategies, such as avoidance, impulsive behaviors, self blame and blaming others (Fletcher et al., 2014). Impulsivity is another core feature of BPD patients, who often show impulsive behavior, such as suicide attempts, binge eating, substance abuse, and unprotected sex (Lieb et al., 2004). Furthermore, unstable and chaotic interpersonal relationship is common in BPD. Patients suffer from serious influences on psychosocial functioning issues (Skodol et al., 2002). In addition, dissociation is a common phenomenon in BPD patients when experiencing stress, including unstable self-perception, disruptions in memory, consciousness, attention etc. (Winter and Schmahl, 2014). Previous studies have shown the prevalence rate of BPD to be between 0.7 and 5.9% in the general population (Coid et al., 2006; Stinson et al., 2008), 15 and 25% in psychiatric inpatients and 10% in psychiatric outpatients (Leichsenring et al., 2011). The variability in prevalence rates may be due to the use of different instruments and assessments.

At present, there are several instruments with proven psychometric properties to assess BPD-related symptoms and screen for BPD. Among these instruments, the Zanarini Rating Scale for Borderline Personality Disorder is a DSM-IV-based clinician-rated scale for assessing borderline symptom from a psychopathology perspective (Zanarini, 2003). The Borderline Personality Disorder Severity Index is a semi-structured interview instrument to evaluate the severity of borderline symptoms (Arntz et al., 2003), and the Clinical Global Impression scale for BPD patients was designed to assess severity and post-intervention changes in patients with BPD (Pérez et al., 2007). In addition to clinically administered instruments, there are several self-reported scales for assessing the symptoms and severity of BPD. For example, the Borderline Evaluation of Severity over Time measures the severity of BPD (Pfohl et al., 2009), and the Borderline Symptom List (BSL), can quickly evaluate typical symptoms of BPD (Bohus et al., 2001). Compared with clinician-interviewed instruments, self-rated scales are more convenient and efficient, as they do not require clinical experts to carry out assessment.

The original version of the BSL (BSL-95) includes 95 items based on diagnosis according to the DSM-IV (Bohus et al., 2001). Each item is scored on a 5-point Likert scale, ranging from 0 (none) to 4 (very strong). A Factor analysis revealed seven factors, including self-perception, emotion regulation, autoaggression, dysthymia, social isolation, intrusions and hostility (Bohus et al., 2001, 2007). While the BSL-95 is a reliable and valid instrument, it is time consuming due to an abundance of items (Bohus et al., 2007; Glenn et al., 2009). Therefore, a shortened version of the BSL (BSL-23) was developed by Bohus et al. (2009) to reduce participant burden. The BSL-23 has been reported to possess good psychometric properties in several languages (Glenn et al., 2009; Soler et al., 2013; Nicastro et al., 2016). The BSL-23 and the BSL-95 are highly correlated, and the BSL-23 has been shown to be more sensitive to changes in symptoms after Dialectical Behavior Therapy than the BSL-95 (Bohus et al., 2009). In addition, the BSL-23 effectively discriminates BPD from other psychiatric disorders, such as attention-deficit hyperactivity disorder and post-traumatic stress disorder (Bohus et al., 2009; Nicastro et al., 2016). Due to differences in cultural backgrounds, it is necessary to examine the psychometric properties of the BSL-23 in the Chinese population, such that the credibility and effectiveness of the tool can be measured for research in China.

Prior findings have indicated that the BSL-23 is highly correlated with tests that measure depression and impulsiveness (Soler et al., 2013; Nicastro et al., 2016). In addition, childhood trauma and attachment style have previously been shown to be central characteristics of BPD (Herman et al., 1989; Hooley and Wilson-Murphy, 2012), about 60–80% BPD patients reported traumatic experiences in childhood, which could contribute to insecure attachment (Bassett, 2012). Therefore, the current study examined the correlations between the BSL-23 and other psychological scales that assess depression, impulsiveness, childhood trauma and attachment style. As the Chinese version of the Personality Diagnostic Questionnaire-4+ (PDQ-4+) has shown adequate psychometric properties to estimate personality disorders (Yang et al., 2002), the borderline subscale of PDQ-4+ was used to screen for BPD in the current study.

## Materials and Methods

### Participants

Participants were undergraduate students (*n* = 570) recruited from two large universities in Guangdong and Hunan province of China through convenience sampling and clinic outpatients (*n* = 323) from the psychological clinic of Second Xiangya Hospital. The clinical patients had diagnoses of depressive disorder, borderline personality disorder, anxiety disorder, schizophrenia, or other psychiatric disorders diagnosed by trained clinicians. The socio-demographic and clinical data of the two samples in detail were presented in **Table 1**.

**Table 1:** Socio-demographic characteristics of samples.

Characteristic description		Undergraduate sample (*n* = 570)	Clinical sample (*n* = 323)	Chi-Square/t	*p*
Gender, n(%)	Male vs. female	153 vs. 417(26.84 vs. 73.16)	139 vs. 184(43.03 vs. 56.97)	24.56	<0.001
Age, Mean ± SD(range)	Years	20.97 ± 1.01(17–26)	25.85 ± 9.49(16–64)	-9.21	<0.001
Diagnosis, n(%)	MDD	/	85(26.32)	/	
	BPD	/	83(25.70)	/	
	Anxiety disorder	/	54(16.72)	/	
	Schizophrenia	/	48(14.86)	/	
	Other	/	53(16.40)	/	
Scales, Mean ± SD(range)	BSL-23	11.82 ± 11.86(0–62)	31.49 ± 22.72(0–92)	-14.48	<0.001
	PDQ-4+ – borderline	2.08 ± 2.00(0–9)	4.09 ± 2.51(0–9)	-12.27	<0.001
	CESD	36.62 ± 9.01(20–66)	49.82 ± 13.02(20–80)	-15.98	<0.001
	BIS-11	64.70 ± 8.58(41–94)	70.44 ± 10.27(43–108)	-8.20	<0.001
	CTQ	38.12 ± 9.27(28—80)	54.05 ± 11.66(32–108)	-20.55	<0.001
	ASQ – Insecure	105.09 ± 17.06(49–192)	114.58 ± 19.36(40–169)	-7.29	<0.001

*MDD, major depressive disorder; BPD, borderline personality disorder; other, other psychiatric disorder, such as somatoform disorder, obsessive–compulsive personality, stress related disorders; BSL-23, borderline Symptom List-23; PDQ-4+ – borderline, personality diagnose questionnaire – borderline subscale; CESD, center for epidemiologic studies depression scale; BIS-11, Barratt impulsiveness scale, 11th version; CTQ, childhood trauma questionnaire; ASQ, attachment style questionnaire.*

Test-retest reliability was determined by a separate sample of 93 undergraduate students (24.70% male and 75.30% female; age range: 20–24; *M*_age_ = 21.52, *SD* = 0.69) who completed the BSL-23 twice (separated by 2 weeks).

All study procedures were approved by the Ethics Committee of Central South University of Second Xiangya Hospital. All participants signed an informed consent.

### Instruments

#### Borderline Symptom List (BSL-23)

The BSL-23 is a self-reported questionnaire that measures the severity of BPD symptomatology (Bohus et al., 2009). Each item of the BSL-23 is scored on a 5-point Likert scale, ranging from 0 (none) to 4 (very strong). The total score is within the range of 0–92. Higher scores indicate more sever borderline symptoms. Previous studies revealed a single factor structure for the BSL-23 (Bohus et al., 2009; Soler et al., 2013; Nicastro et al., 2016). The forward and back translation of the Chinese version of the BSL-23 was conducted by bilingual psychiatric experts, independently. No items were removed or changed during the translation. The final 23 items in Chinese proved comprehensible to Chinese participants in a preliminary study.

#### Personality Diagnostic Questionnaire-4+ (PDQ-4+)-Borderline Subscale

The PDQ-4+ is a self-rated personality disorder questionnaire including 12 subscales corresponding to 12 types of personality disorders (Hyler et al., 1992; Yang et al., 2002). The borderline subscale comprises 9 items each scored with 0 or 1. The total score, therefore ranges from 0 to 9. A total score ≥ 5 indicates a borderline personality disorder. The Chinese version of the PDQ-4+ has previously been shown to have acceptable psychometric properties (Yang et al., 2002). The current study adopted the borderline subscale of the PDQ-4+ to screen a BPD positive group (score of PDQ-4+ -Borderline subscale ≥ 5) and the BPD negative group (score of PDQ-4+ -Borderline subscale < 5). In this study, the Cronbach’s α of PDQ-4+ -Borderline subscale was 0.73 in undergraduate sample and 0.77 in clinical sample.

#### Center for Epidemiologic Studies Depression Scale (CES-D)

The CES-D is a 20-item self-reported questionnaire evaluating depressive symptoms during the previous week (Demirchyan et al., 2011). Each item is scored on a 4-point Likert scale, ranging from 1 (never) to 4 (very often). The total score of the CES-D, therefore ranges from 20 to 80. The psychometric properties of the CES-D were suitable in Chinese participants (Wang et al., 2013). The Cronbach’s α of CES-D was 0.83 in undergraduate sample and 0.88 in clinical sample in this study.

#### Barratt Impulsiveness Scale, 11th Version (BIS-11)

The BIS-11 measures impulsivity (Barratt and Patton, 1983) and includes 30 items, each on a 4-point Likert scale, from 1 (never) to 4 (always). The total score ranges from 30 to 120. The BIS-11 contains three subscales (motor impulsivity, cognitive impulsivity, lack of planning). The Chinese version of the BIS-11 has shown good reliability and validity (Yao et al., 2007). In our samples, the Cronbach’s α of BIS-11 was 0.69 in undergraduates and 0.68 in patients, respectively.

#### Childhood Trauma Questionnaire (CTQ)

The CTQ includes 28 items that assess childhood abuse and neglect experiences (Bernstein et al., 2003). Each item is scored on a 5-point scale, ranging from 1 (never) to 5 (always). The total score ranges from 28 to 140. The CTQ contains five subscales: emotional abuse, physical abuse, sexual abuse, emotional neglect and physical neglect. The Chinese version of the CTQ has been shown to have adequate psychometric properties (Li et al., 2014). In this study, the Cronbach’s α of CTQ was 0.65 in undergraduate sample and 0.70 in clinical sample.

#### Attachment Style Questionnaire (ASQ)

The ASQ is a 40-item self-reported questionnaire assessing attachment style (Feeney et al., 1994). Each item is on a 6-point scale rating from 1 (totally disagree) to 6 (totally agree). The total score of the ASQ ranges from 40 to 240. The ASQ consists of five subscales: confidence, discomfort, relationships as secondary, need for approval, and preoccupation. The current study used the combined total of the last four subscales, which has previously been shown to reflect insecure attachment style (Hazan and Shaver, 1987). Scores range from 32 to 192 (Yi et al., 2012). The ASQ has been shown to have good reliability and validity in a Chinese population (Yi et al., 2012). In this study, the Cronbach’s α of ASQ was 0.85 in undergraduate sample and 0.82 in clinical sample.

### Data Analysis

Data were analyzed using IBM SPSS Statistics 20.0 and AMOS 17.0. Listwise deletion was used to handle the missing data in analysis. The results of skewness and kurtosis of each BSL-23 item indicated near normal distribution for most items, except items 5, 7, 11, 18, and 19.

First, a Confirmatory Factor Analysis (CFA) was performed to examine the one-factor structure of the BSL-23. The maximum likelihood (ML), a common method of estimation within CFA (Flora and Curran, 2004), was run. Several fit indexes were performed, including the parsimonious goodness of fit index (PGFI), the incremental fit index (IFI), the Tucker–Lewis index (TLI), the comparative fit index (CFI) and the root mean square error of approximation (RMSEA) with a 90% confidence interval (CI). Generally, PGFI ≥ 0.500, IFI ≥ 0.900, TLI ≥ 0.900, CFI ≥ 0.900, and RMSEA ≤ 0.080 is considered acceptable (Browne and Cudeck, 1992; Hu and Bentler, 1999). To provide more information for the construct validity of the BSL-23, we also randomly split each sample in half and performed Exploratory Factor Analysis (EFA) and CFA in both samples, and the results of EFA and CFA with subsamples were shown in Supplementary Material.

Second, Cronbach’s alpha, Omega, split-half reliability and the M_IC_ of the BSL-23 were calculated for both the undergraduate students and the clinical population. The test-retest stability was estimated in an undergraduate sub-sample (see above). The correlation coefficient was evaluated between the BSL-23 and the PDQ-4+-borderline subscale to assess criterion-related validity. The correlations between the BSL-23 and the CES-D, the BIS-11, the CTQ and the ASQ were also computed to assess convergent validity.

Third, to further assess criterion-related validity, the two samples were divided into two groups (BPD screening-positive group and BPD screening-negative group) according to the score on the PDQ-4+-borderline subscale (see above for cut-off). An independent samples *t*-test was performed to compare the difference in BSL-23 score between the two groups. Cohen’s *d* was calculated to estimate the effect sizes (Cohen, 1992).

Finally, the differences in BSL-23 scores between undergraduate students and clinical patients were examined by an independent samples *t*-test.

## Results

### Confirmatory Factor Analysis

The fit indexes values obtained by the one-factor model were good both in the undergraduate student sample and in the clinical sample (**Table 2**). The PGFI values were greater than 0.500, and the values of the IFI, the TLI and the CFI were all greater than 0.900 in both samples, indicating that the fitness of a one-factor model was acceptable. Moreover, values of the RMSEA were less than 0.080, suggesting a reasonable error approximation. The factor loading of all items of the BSL-23 in both samples ranged from 0.45 to 0.84 (**Table 3**).

**Table 2:** The fit indexes of the CFA in the undergraduate sample and clinical sample.

							RMSEA 90%CI	
	χ^2^/df	PGFI	IFI	TLI	CFI	RMSEA	LO90	HI90
Undergraduate students	3.440	0.675	0.922	0.905	0.922	0.065	0.060	0.071
Clinical patients	2.923	0.650	0.935	0.922	0.935	0.077	0.070	0.084

*χ^2^, Chi-square; df, degrees of freedom; PGFI, parsimonious goodness-of-fit index; IFI, incremental fit index; TLI, Tucker– Lewis index; CFI, comparative fit index; RMSEA, root-mean-square error of approximation; LO90 and HI90 indicate lower and upper end of the 90% confidence interval of the RMSEA.*

**Table 3:** The factor loading of each item of BSL-23 in the undergraduate sample and clinical sample.

	Undergraduate sample	Clinical sample		Undergraduate sample	Clinical sample
BSL01	0.45	0.65	BSL13	0.67	0.61
BSL02	0.61	0.78	BSL14	0.75	0.77
BSL03	0.47	0.67	BSL15	0.57	0.66
BSL04	0.63	0.74	BSL16	0.63	0.71
BSL05	0.59	0.77	BSL17	0.64	0.77
BSL06	0.64	0.64	BSL18	0.67	0.75
BSL07	0.54	0.75	BSL19	0.58	0.76
BSL08	0.58	0.77	BSL20	0.71	0.72
BSL09	0.57	0.77	BSL21	0.77	0.84
BSL10	0.50	0.67	BSL22	0.69	0.83
BSL11	0.71	0.81	BSL23	0.70	0.81
BSL12	0.70	0.78			

### Reliability

In the undergraduate sample, the Cronbach’s alpha was 0.93, the Omega coefficient was 0.95, the split-half coefficient was 0.89, and the M_IC_ was 0.38. The test-retest stability within 2 weeks was 0.62. For the clinical sample, the Cronbach’s alpha was 0.97, and the Omega coefficient was 0.97, the split-half coefficient was 0.96 and the M_IC_ was 0.56.

### Correlations Between BSL-23 and Other Psychological Scales

There were significant correlations between the BSL-23 and the borderline subscale of the PDQ-4+, the CES-D, the BIS-11, the CTQ, and the insecure subscale of the ASQ in both the undergraduate (*r* = 0.35–0.60) and clinical (*r* = 0.35–0.70) samples (**Table 4**).

**Table 4:** Correlations between scores on BSL-23 and psychological scales in the undergraduate sample and clinical sample.

	BSL-23	
	Undergraduate students	Clinical patients
PDQ-4+ - borderline subscale	0.50^∗∗^	0.70^∗∗^
CESD	0.60^∗∗^	0.69^∗∗^
BIS-11 Total score	0.35^∗∗^	0.54^∗∗^
CTQ Total score	0.43^∗∗^	0.35^∗∗^
ASQ – Insecure subscale	0.47^∗∗^	0.52^∗∗^

*PDQ-4+ – borderline subscale, personality diagnostic questionnaire – borderline subscale; CESD, center for epidemiologic studies depression scale; BIS-11, Barratt impulsiveness scale, 11th version; CTQ, childhood trauma questionnaire; ASQ, attachment style questionnaire. ^∗∗^p < 0.01.*

### Differences Between the BPD Screening-Positive and Screening-Negative Groups on BSL-23 Scores

Among the undergraduate sample, there were 85 participants (15%) in the BPD screening-positive group and 465 undergraduates in the BPD screening-negative group (85%). An independent samples *t*-test found a significantly higher BSL-23 score in the positive group compared to the negative group (*p* < 0.01). Among the clinical sample, there were 146 patients (46%) in the BPD screening-positive group and 174 patients (64%) in the BPD screening-negative group. The patients in the positive group scored significantly higher than the patients in the negative group on the BSL-23 (*p* < 0.01) (**Table 5**).

**Table 5:** BSL-23 total score in the BPD screening-positive and BPD screening-negative groups within both the undergraduate sample and clinical sample.

	BPD positive (*n*)	BPD negative (*n*)	*p*	Cohen’s *d*
Undergraduate students	22.93 ± 15.15 (85)	9.72 ± 9.93 (465)	<0.001	1.03
Clinical patients	45.69 ± 20.80 (146)	19.48 ± 16.71 (174)	<0.001	1.39

### Differences Between Undergraduate Students and Clinical Patients on BSL-23 Scores

Clinical patients scored significantly higher on the BSL-23 than the undergraduate students (*p* < 0.01, Cohen’s *d =* -1.09).

## Discussion

The BSL-23 has previously been shown to be a valid and efficient tool in the measurement of the severity of borderline symptoms (Soler et al., 2013; Nicastro et al., 2016). Compared to the initial version of the questionnaire, the BSL-23 not only has comparable psychometric properties, but also saves time and remarkably reduces the burden on participants. In agreement with prior studies (Bohus et al., 2009; Soler et al., 2013; Nicastro et al., 2016), the current study, supported the one-factor structure model of the BSL-23 in both an undergraduate student sample and a clinical sample.

In the present study, the Cronbach’s α of the BSL-23 was 0.93 in undergraduates, and 0.97 in clinical patients, both in agreement with prior studies reporting a range of 0.94 – 0.97 (Bohus et al., 2009; Soler et al., 2013; Nicastro et al., 2016). In addition, the Split-Half coefficients were high in both the undergraduate sample (0.89) and the clinical sample (0.96). The M_IC_ of the undergraduate sample was within the optimal range (0.10–0.50), as suggested by Brigges and Cheek (1986), while the M_IC_ of the clinical sample was a just over 0.50. Overall, the results of the current study support a high internal consistency of the Chinese version of the BSL-23.

Prior studies have shown a test-retest reliability within 1 week of *r* = 0.73 – 0.84 (Bohus et al., 2009; Soler et al., 2013; Nicastro et al., 2016), supporting the stability of BPD severity. However, the test-retest reliability within 2 weeks in the current study was less favorable (*r* = 0.62). The discrepancy is likely due to a longer test-retest time interval and the relatively smaller sample size in the current study.

There were significant correlations between the BSL-23 and the borderline subscale of the PDQ-4+ in both the undergraduate sample (*r* = 0.50) and the clinical sample (*r* = 0.70). While the BPD screening-positive group and BPD screening-negative group were determined by the PDQ-4+ BPD subscale, the two groups differed significantly in BSL-23 scores, further supporting reasonable criterion-related validity.

Previous studies have found that patients with BPD have a higher level of depression and anxiety than the general population (Luca et al., 2012; Friborg et al., 2014; Gremaud-Heitz et al., 2014). In the current study, we found that scores on the Chinese version of the BSL-23 correlated strongly with scores on the CES-D in both the undergraduate and the clinical samples, which was consistent with previous findings that scores on the BSL-23 correlate strongly with depression assessed by the Beck Depression Inventory (Bohus et al., 2007, 2009; Soler et al., 2013; Nicastro et al., 2016). Moreover, scores on the BSL-23 were highly correlated with the BIS-11 in both samples. These results indicate that BPD patients are characterized as more impulsive, as previous studies have shown that impulsivity measured by BIS was positively correlated with BSL-23 score (Soler et al., 2013; Nicastro et al., 2016). In summary, correlations between BPD symptoms measured by the BSL-23 and depression and impulsivity are consistent with the idea that BPD patients have a greater incidence of affective symptoms.

BSL-23 scores exhibited moderate to high correlations with CTQ scores in both the undergraduate sample and the clinical sample, suggesting that individuals with more serious borderline symptoms may have experienced more abuse and neglect in childhood. These findings are consistent with prior studies showing that 70% of BPD patients report childhood trauma experiences (Ball and Links, 2009; Blasczyk-Schiep and Jaworska-Andryszewska, 2014). Moreover, scores on the insecure attachment subscale showed a strong positive relationship with BSL-23 scores, suggesting that individuals with greater borderline symptoms also have a higher level of insecure attachment style, providing further support for Agrawal’s conclusion that insecure attachment style strongly correlates with BPD regardless of measurement methods (Agrawal et al., 2004). A recent study found that insecure attachment partially mediates the relationship between childhood trauma and borderline symptoms (Baryshnikov et al., 2017). It may be that childhood trauma leads to insecure attachment, which consequently contributes to borderline symptoms. Further studies examining trauma, insecure attachment and borderline symptoms in one model are needed to clarify the relationship (e.g., mediating or moderator) among variables.

Lastly, BSL-23 scores were significantly greater among the clinical patients compared to the undergraduate students, indicating that the BSL-23 is an effective tool in the evaluation of BPD symptom severity. This finding provides support for the application of the BSL-23 in the Chinese population, both in the general population and in the clinical population.

In summary, this study confirmed the single factor structure and good psychometric properties of the BSL-23 in Chinese population, which provides an efficient and standardized instrument for Chinese clinicians to estimate the severity of BPD symptoms. With the BSL-23, clinicians can monitor the severity of BPD symptoms in the process of treatment, and assess the treatment efficacy in BPD patients, which will be helpful for further choice of intervention and therapy. While our findings supported the reliability and validity of the Chinese version of the BSL-23, some limitations should be considered. First, the homogeneity of the two samples in gender and age is not applicable, which limit the comparability of the two samples. Therefore, further studies are needed to reexamine the results we got in this study with two socio-demographic homogeneous samples (non-clinical and clinical sample). Second, the global measurement of dissociation of BPD was not included in this study. For dissociation is one of important symptoms in BPD, the relationship between dissociative symptoms and the BSL-23 should be examined in further research to provide comprehensive support for the BSL-23. Third, the clinical group consisted of a variety of psychological disorders, not just BPD diagnosis. Since BSL-23 is a tool for assessing the severity of BPD symptoms, examining its reliability and validity in patients with BPD will be more accurate and convincing, which might also contribute to clarify whether the Chinese version of the BSL-23 can distinguish BPD from other psychological disorders. Fourth, the patients in the current study did not receive psychotherapeutic intervention, and as such whether the Chinese version of the BSL-23 is sensitive to treatment changes cannot be discerned. Future studies are needed to compare BSL-23 scores between patients with BPD and patients with other psychological disorders, and to examine the sensitivity of BSL-23 to treatment effects in Chinese BPD patients.

## Conclusion

The BSL-23 is a self-rated scale that was developed to estimate the severity of BPD symptomatogy. In the current study, the one-factor structure of Chinese version of the BSL-23 was confirmed both in undergraduate students and in clinical patients. The Chinese version of the BSL-23 exhibited good internal consistency, criterion-related validity and high convergent validity. In addition, the BSL-23 was proven highly sensitive in the discrimination between BPD screening-positive individuals and BPD screening-negative individuals. Moreover, The BSL-23 was shown to have the capacity to discriminate BPD severity between a patient sample and undergraduate students. In all, the Chinese version of the BSL-23 shows satisfactory psychometric properties, offering an efficient and practical instrument for clinicians to assess borderline symptomatogy in Chinese population.

## Author Contributions

JY designed the study. XL, MZ, QZ, and YL contributed to the data collection. HY analyzed the data and drafted the manuscript. MJ and JY revised the manuscript.

## Conflict of Interest Statement

The authors declare that the research was conducted in the absence of any commercial or financial relationships that could be construed as a potential conflict of interest.
