# Supplementary material for: Psychometric Properties of the Chinese Version of the Brief Borderline Symptom List in Undergraduate Students and Clinical Patients
Source: Front Psychol. 2018 Apr 27;9:605. doi: 10.3389/fpsyg.2018.00605 (PMC5934523; doi:10.3389/fpsyg.2018.00605)
Supplement: Supplementary file 1 [file Presentation_1.pdf]

Supplementary Material

Psychometric Properties of the Chinese Version of the Brief Borderline Symptom List in Undergraduate Students and Clinical Patients

Methods

We randomly split each sample in half and performed Exploratory Factor Analysis (EFA) and Confirmatory Factor Analysis(CFA) in subsamples. In the undergraduate sample, there were 277 individuals in the subsample for EFA, and 284 in the subsample for CFA. In the clinical sample, the subsample for EFA included 164 patients, and 147 patients were in the subsample for CFA. There were no significant differences of age and gender between the two subsamples of undergraduates (*ps* > 0.05), neither the clinical subsamples (*ps* > 0.05).

EFA Results

In the undergraduate subsample, the KMO measure of sampling adequacy was high (0.918) and Bartlett's test of sphericity was significant (p<.001), indicating that our undergraduate data was suited for factor analysis. The principal components analysis showed three components with eigenvalues greater than 1 (9.943, 2.020, 1.696) cumulatively explaining 57.646% of total variance, while the scree plot indicated one factor (**FIGURE S1**) explaining 43.229% variance. In the clinical subsample, the value of KMO test was 0.950 and the Bartlett's test of sphericity was significant (p<.001), indicating that the clinical data was appropriate for factor analysis. Although principal components analysis showed three components with eigenvalues greater than 1 (13.468, 1.404, 1.063) cumulatively explaining 69.286% of total variance, the scree plot indicated one factor (**FIGURE S2**) explaining 58.557% variance. Our EFA results partly supported the previous one factor structure model.

CFA Results

We performed the CFA in both the undergraduate subsample and the clinical subsample. The Chi square test results and incremental indexes of both subsamples supported the psychometric properties. The PGFI values were greater than 0.500, and

the values of the IFI, the TLI and the CFI were all greater than 0.900 in both samples, indicating favorable goodness of fit of one-factor model. Moreover, values of the RMSEA were less than 0.080, suggesting that the error approximations were acceptable, see **Table S1**. All the CFA results in subsamples were in line with the results of CFA in whole samples.

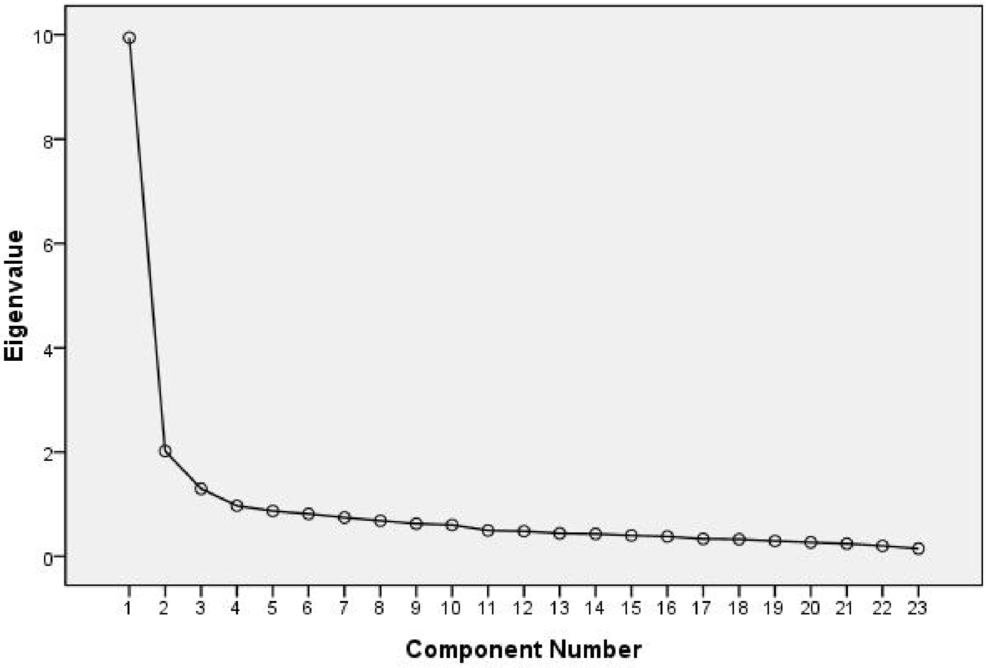

FIGURE S1. The scree plot of BSL-23 in undergraduate subsample (n=277)

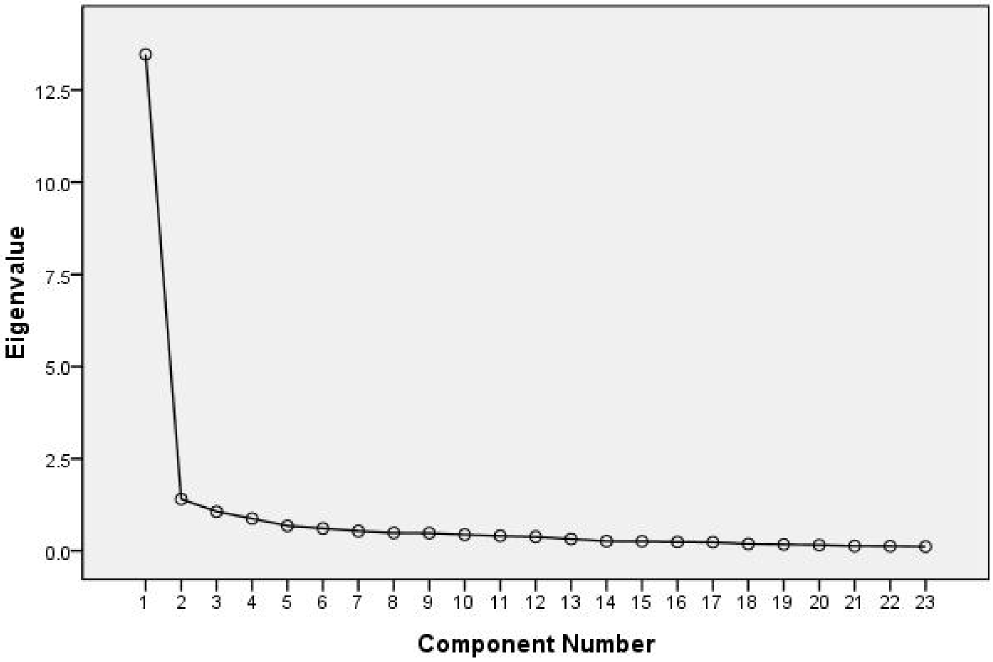

FIGURE S2. The scree plot of BSL-23 in clinical subsample (n=164)

TABLE S1. The fit indexes of the CFA in the undergraduate and clinical subsamples

							RMSEA 90%CI	
	$\chi2$/df	PGFI	IFI	TLI	CFI	RMSEA	LO90	HI90
Undergraduate Students	2.097	0.686	0.919	0.904	0.918	0.062	0.054	0.070
Clinical Patients	1.868	0.613	0.940	0.926	0.939	0.077	0.065	0.089

Note: $\chi2$ =Chi-square; df = degrees of freedom; PGFI = parsimonious goodness-of-fit index; IFI = incremental fit index; TLI = Tucker– Lewis index; CFI = comparative fit index; RMSEA = root-mean-square error of approximation; LO90 and HI90 indicate lower and upper end of the 90% confidence interval of the RMSEA.
